# Building adolescents’ resilience: teachers’ support, perceived cooperative and competitive climates, and mindset

**DOI:** 10.3389/fpsyg.2026.1897482

**Published:** 2026-07-29

**Authors:** Hui Wang, Anqi Peng, Meagan M. Patterson

**Affiliations:** 1 Department of Psychology, McKendree University, Lebanon, IL, United States; 2 T. Denny Sanford Harmony Institute, Arizona State University, Tempe, AZ, United States; 3 Department of Educational Psychology, University of Kansas, Lawrence, KS, United States

**Keywords:** adolescents, cooperation and competition, mindset, resilience, teacher support

## Abstract

**Introduction:**

Resilience helps adolescents persist in challenging situations and recover from setbacks.

**Methods:**

This study examined how several school-related contextual factors (i.e., perceived cooperative and competitive climate and teachers’ academic and emotional support) and one individual factor (i.e., mindset) related to three components of resilience (i.e., self-efficacy, perseverance, and sense of meaning in life) among a sample of U.S. adolescents, using the Programme for International Student Assessment 2018 data.

**Results:**

Overall, the findings revealed that all examined factors positively predicted adolescents’ resilience. Additionally, while teachers’ support as well as perceived competitive climate positively predicted mindset, perceived cooperative climate was negatively linked to mindset. Moreover, an indirect effect through mindset was observed only for teachers’ academic support.

**Discussion:**

The findings suggest that fostering resilience in adolescents may require a comprehensive approach, emphasizing the role of teachers’ academic support, emotional understanding, a balance between cooperative and competitive learning climates, and promotion of a growth mindset.

## Introduction

Adolescence is a developmental period characterized by various physical, emotional, psychological, and social changes and challenges. During this stage, adolescents may experience rapid physical transformations, intense academic expectations, and stressors related to identity formation and peer relationships, which can in turn threaten overall mental health (Du et al., 2021). Therefore, exploring strategies to protect adolescents from adversity is critical for fostering their psychological well-being and positive development.

Researchers view resilience as a vital protective factor to help adolescents navigate adversity (Anderson and Priebe, 2021; Vinayak and Judge, 2018). By developing resilience skills such as persisting toward goals and seeking meaning in life, adolescents build the inner strength to bounce back from setbacks, adapt to change, and maintain optimism in difficult circumstances. Within the limited existing research, studies have identified both individual and environmental factors that promote adolescents’ resilience (Frisby et al., 2020; Walsh et al., 2020). However, most empirical studies have overlooked the multifaceted nature of resilience and its potential complex interactions with environmental and individual factors. Therefore, grounded in Social Cognitive Theory (SCT), this study examined the predictive power of an individual factor (i.e., mindset) and several school-based environmental factors (i.e., teachers’ academic and emotional support, perceived cooperative and competitive climates), as well as their interactions, in predicting several resilience factors (i.e., self-efficacy, perseverance, and sense of meaning in life) among a sample of U.S. adolescents.

### Resilience

Derived from the Latin word *resilio*, meaning “to jump back,” resilience represents an individual’s capacity to bounce back from adversity and cope with the demands inherent in a specific life stage (Kim and Kim, 2016; Masten and Reed, 2002). Fostering resilience can help adolescents navigate change, develop healthy coping strategies, recover from failures, and maintain mental well-being (Azpiazu Izaguirre et al., 2021; Vinayak and Judge, 2018).

Although there is no consensus on a universal definition of resilience (Anderson and Priebe, 2021), research has consistently demonstrated that resilience is a multidimensional construct encompassing a range of cognitive, behavioral, and motivational capacities. For example, Wagnild and Young (1993) identified several core components of resilience, including perseverance, sense of purposeful life, and self-reliance. Similarly, Zhan (2018) identified related components such as self-efficacy, perseverance, and positive cognition. Other studies have measured resilience using elements such as emotional regulation and goal orientation (Zhao and Li, 2009) and help-seeking (Zhan, 2018), all of which reflect individuals’ capacity to overcome adversity through individual or collective action. Despite this variation in operationalization, three key elements emerge with consistency across the resilience literature: self-efficacy, perseverance, and sense of meaning in life (Cassidy, 2016; Hu and Gan, 2008; Park, 2010; Wagnild and Young, 1993; Zhao and Li, 2009). Thus, these three dimensions were selected as indicators of resilience in the present study, as they represent the most consistently identified and empirically supported components of resilient functioning across diverse samples.

Specifically, self-efficacy refers to the belief in one’s ability to engage in certain activities and perform specific tasks, especially when facing challenging circumstances (Bandura, 1997). Theoretically, self-efficacy constitutes a core component of resilience because it is the primary cognitive mechanism through which individuals believe that they can develop critical skills and find solutions in the face of adversity (Ouweneel et al., 2013). Perseverance describes the ability to continue striving to achieve a goal despite setbacks and failures, representing a commitment and determination to persist through challenges, maintain effort, and adjust strategies when confronting obstacles (Cassidy, 2016; Park et al., 2020). As the behavioral expression of resilience, perseverance captures active, sustained effort when confronting difficulties. Individuals with high levels of perseverance can bounce back quickly from failures and sustain motivation during difficult periods (Eskreis-Winkler et al., 2014). Sense of meaning in life, functioning as a key motivational resource, refers to one’s drive to integrate life experiences into a cohesive and meaningful narrative (Heintzelman and King, 2014; Park, 2010). Individuals who perceive their lives as meaningful and purposeful demonstrate greater resilience against stress and are better able to apply effective coping strategies when confronting adversity (Heintzelman and King, 2014). All of these cognitive, behavioral, and motivational dimensions offer a theoretically coherent and empirically grounded operationalization of resilience for the present study.

Resilience can involve utilizing both personal and social resources, and thus can be influenced by both internal and environmental factors. Drawing on both extant empirical research and Social Cognitive Theory (SCT; Bandura, 2001), resilience varies based on both situational contexts and individual factors, such as knowledge, goals, and outcome expectations. Compared with the research examining the effects of resilience, fewer studies have investigated the complex interactions of factors that predict resilience. Although some studies in educational contexts have investigated the effects of various contextual factors – such as pedagogical approaches, teaching styles, and culturally responsive classroom management (Bondy et al., 2007; Walsh et al., 2020) – and individual cognitive factors (e.g., beliefs, self-confidence; Frisby et al., 2020) on resilience, the existing research has largely overlooked the complex interactions among environmental and individual factors. It is worth noting that although studies have investigated teachers’ teaching styles and management approaches, the nuanced impact of other school-related factors, such as teachers’ academic and emotional support, remain largely unexamined. Additionally, the role of other individual factors, such as mindset, is yet to be well investigated.

### Mindset

Mindset refers to individuals’ beliefs about human attributes, particularly intelligence (Dweck and Yeager, 2019). Individuals who believe intellectual capacities can grow and develop over time are considered to have a growth mindset; those who view intellectual abilities as innate and unchangeable are characterized as having a fixed mindset (Burnette et al., 2013; Dweck and Yeager, 2019). Adolescents with a growth mindset are more likely to embrace challenges, persist in the face of difficulties, and view effort as a key component of goal achievement. In contrast, those with a fixed mindset tend to avoid challenges, give up easily when faced with setbacks, and interpret effort as a sign of incompetence, and are more likely to experience mental health problems (Park et al., 2020; Walker and Jiang, 2022).

Growth mindsets can enhance adolescents’ resilience (Iqbal et al., 2021; Janssen and van Atteveldt, 2023; Yeager and Dweck, 2012). As an example, Janssen and van Atteveldt (2023) examined the protective effects of growth mindset on academic resilience among adolescents during the COVID-19 pandemic. The results suggested that growth mindset directly and indirectly predicted resilience through coping styles. Additionally, Yeager and Dweck’s (2012) review further demonstrated that adolescents who viewed intellectual abilities as malleable rather than fixed demonstrated greater resilience in the face of academic and social challenges, as they exhibited higher achievement during challenging school transitions and higher course completion rates. However, previous empirical research has paid little attention to the multidimensional nature of resilience when exploring the effects of mindset, which may miss crucial nuances in how mindset impacts different aspects of resilience.

A relevant body of existing research has examined mindset as a mediator between aspects of the classroom environment and students’ attitudes or behaviors. For example, various researchers have argued that teacher practices, such as classroom instruction and feedback on student work, shape students’ mindsets, which in turn influence students’ learning-related behaviors such as persistence and goal-setting (Dweck and Yeager, 2019; Laine and Tirri, 2023). This approach is consistent with the Social Cognitive Theory framework (Bandura, 2001) in which the environment, cognitions, and behaviors all relate to one another, and cognitions provide an important bridge by which previous experiences shape current and future behaviors.

### Teachers’ support

Researchers have broadly defined teachers’ support as a construct including both direct and indirect resources that teachers provide to enhance students’ academic achievement (Wentzel, 1998). Teacher support includes both academic support and emotional support (Liu et al., 2018; Patrick et al., 2011). Teachers’ academic support refers to students’ perceptions of receiving help to enhance their learning, understanding, and academic performance. Teachers’ emotional support reflects students’ sense that teachers care about them as distinct individuals.

A large body of research suggests that teachers’ academic and emotional support have beneficial impacts on adolescents’ academic performance and well-being (Chen, 2005; Kikas and Mägi, 2017; Tennant et al., 2014). For example, Chen (2005) examined how adolescents’ self-perceived academic support from parents, teachers, and peers related to their academic achievement. The results showed that all three types of academic support positively predicted achievement through academic engagement, with teachers’ academic support being the strongest contributor. In another study, Tennant et al. (2014) found that teachers’ emotional support was positively related to adolescents’ academic outcomes and personal adjustment and negatively associated with school problems, internalizing problems, and hyperactivity.

In recent years, the relations between teachers’ support and students’ growth mindset have attracted attention from scholars. A few studies have revealed that teachers’ support can facilitate students’ growth mindset in secondary school settings (Kroeper et al., 2022; Vestad and Bru, 2024; Yu et al., 2022). For example, Yu et al. (2022) found that teachers’ autonomy-supportive practices, especially guided inquiry practices, represented an important context for students’ growth mindset development. Vestad and Bru (2024) also found that teachers’ support can facilitate students’ growth mindset, which in turn promotes students’ academic engagement and achievement. Although these findings highlight the influence of teachers’ academic support for promoting growth mindset, the existing literature has overlooked the possible role of teachers’ emotional support.

When examining teachers’ support and students’ resilience, most studies have conceptualized teachers’ support and resilience as unidimensional constructs rather than investigating their various subdimensions in relation to each other (Guo et al., 2020; Kang et al., 2024). For example, Guo et al. (2020) investigated how teachers’ support was associated with mental well-being among Chinese adolescents. The findings indicated that resilience mediated the link between teachers’ support and adolescents’ mental well-being. However, teachers’ academic and emotional support were integrated into a unidimensional construct, which makes it unclear what the specific impact of each was.

### Perceived cooperative and competitive climates

Schools are settings where adolescents simultaneously navigate cooperative and competitive relationships with their peers (Li, 2017; Ooi and Cortina, 2023; Williams and Sheridan, 2010). For example, students might cooperate in the context of group projects or peer tutoring but feel a sense of competition with peers in the college admissions process.

Adolescence is a time in which opportunities for both competition and cooperation increase meaningfully. Adolescents compare themselves to peers across multiple dimensions, including academic performance, social acceptance, and athletic competence (Harter, 2012). Furthermore, secondary schools often reinforce such comparisons through high stakes testing and competitive extracurricular activities (e.g., varsity sports, debate leagues). At the same time, adolescents’ advances in social skills and executive function abilities allow for effective collaboration (Landry et al., 2009), and many competitive activities also involve cooperation and teamwork (e.g., Levy, 2016).

A wide range of literature has demonstrated the benefits of cooperative educational climates for learning engagement, empathy, and school belonging (Chen et al., 2025; Lubicz-Nawrocka, 2019; Williams and Sheridan, 2010). However, cooperative environments may not always produce the desired positive outcomes; when Liow (2022) conducted an experimental study to explore the role of collaborative team learning on students’ growth mindset, the results surprisingly showed that students in the collaborative learning group showed decreases in growth mindset.

The impacts of competitive climates are even less clear. Some studies have found that highly competitive environments can increase adolescents’ stress and anxiety and contribute to problems with peers (Lätsch, 2017), whereas others argue that competition can facilitate emotional regulation skills, communication abilities, mastery-approach goals, and achievement (Chen and Chiu, 2016; Chen et al., 2025; Kim et al., 2023). While little research has directly focused on the link between competition and mindset, the results of a meta-analysis revealed that individuals who primarily concentrate on outperforming others may be less likely to adopt a growth mindset, as they prioritize proving their current abilities over learning and improvement (Burnette et al., 2013).

Research examining the relations between cooperative and competitive environments and adolescents’ resilience remains limited. Regarding cooperative environments, Baldrighi et al. (2011) conducted a case study to examine how cooperative learning experiences foster resilience among secondary school students. The findings suggested that when key components of cooperation were provided (e.g., trusting relationships, effective communication), students were found to be more resilient. However, the relation between competitive environments and resilience remains largely unexplored. Thus, this gap calls for more research investigating the connections between perceived cooperative and competitive environments and resilience.

### The present study

Although existing research has demonstrated that resilience is shaped by both contextual and individual factors (Frisby et al., 2020; Walsh et al., 2020), critical gaps remain in understanding the underlying mechanisms driving the complex interactions among these variables. First, a significant portion of the existing literature has positioned resilience as a predictor and overlooked factors that contribute to its development. Within the limited research examining predictors of resilience, the distinct roles of teachers’ academic and emotional support and perceived competitive climate remain unclear. Additionally, the interrelations among predictors remain uncertain. Particularly, although studies have demonstrated the critical impact of mindset on shaping resilience among adolescents, the indirect effects of teachers’ support, especially emotional support, as well as the perceived cooperative and competitive climate on resilience through mindset have yet to be well understood. Furthermore, previous empirical research has treated resilience as a unidimensional construct rather than considering its multifaceted nature, which leads to an incomplete understanding of the underlying mechanisms of resilience. Thus, grounded in SCT, this study examined the interactive role of teachers’ academic and emotional support, perceived cooperative and competitive climates, and mindset in shaping resilience among U.S. adolescents. Accordingly, the following research questions were examined:

RQ1: Do teachers’ academic and emotional support directly predict mindset and the three components of resilience (i.e., self-efficacy, perseverance, and sense of meaning in life)?

RQ2: Do perceived cooperative and competitive climates directly predict mindset and the three components of resilience?

RQ3: Does mindset directly predict the three components of resilience?

RQ4: Do teachers’ academic and emotional support and perceived cooperative and competitive climates indirectly impact the three components of resilience via mindset?

## Methods

### Participants

The data was drawn from the Organization for Economic Co-operation and Development (OECD) Programme for International Student Assessment (PISA) 2018. PISA is an international large-scale assessment project with the aim of evaluating educational systems and students’ academic performance. In the present study, the participants (*N* = 4838; 49.1% females, 50.9% males) were 15-year-old (*M* = 15.84, *SD* = 0.29) U.S. students who participated in the PISA 2018 cycle. The sample was diverse in terms of race and ethnicity: 43.34% White, 25.24% Hispanic/Latino, 15.89% Black/African American, 7.59% multiracial, 5.87% Asian, and 0.97% from other races. Most participants spoke English at home (83.8%); others reported speaking Spanish (10.7%) or another language (4.5%).

### Measures

All the variables of interest were measured with items in the PISA Student Questionnaire (OECD, 2018).

#### Teachers’ academic support

Teachers’ academic support was measured with four items (e.g., “The teacher shows an interest in every student’s learning.”). Participants were asked to report how often the behaviors described in each item occurred during their English classes from *every lesson* (1) to *never or hardly ever* (4). All items were reverse-scored, with higher scores indicating higher levels of teacher academic support. The Cronbach’s alpha in this sample was 0.90.

#### Teachers’ emotional support

Teachers’ emotional support was assessed by three items (e.g., “The teacher made me feel confident in my ability to do well in the course.”). Students were asked to indicate their agreement with each item in their English classes using a scale from 1 (*strongly disagree*) to 4 (*strongly agree*). Higher scores indicated greater emotional support. The Cronbach’s alpha in this sample was 0.88.

#### Perceived cooperative climate

Perceived cooperative climate at the participant’s school was assessed by four items (e.g., “Students seem to value cooperation.”), with each rated ranging from 1 (*not at all true*) to 4 (*extremely true*). Higher scores reflected participants’ perception of a more cooperative environment. The Cronbach’s alpha in this sample was 0.91.

#### Perceived competitive climate

Perceived competitive climate at the participant’s school was also assessed by four items (e.g., “Students seem to value competition.”), with each rated ranging from 1 (*not at all true*) to 4 (*extremely true*). Higher scores indicated that participants perceived the environment as more competitive. The Cronbach’s alpha in this sample was 0.83.

#### Mindset

A single item was used to measure participants’ mindset (i.e., “Your intelligence is something about you that you can’t change very much.”), with this item rated on a 4-point Likert scale ranging from 1 (strongly disagree) to 4 (strongly agree). This item was reverse-scored, with a higher score indicating a stronger growth mindset. Single-item measures of growth mindset have been shown in prior studies to have strong psychometric properties. Specifically, of all three items in Dweck’s (2006) growth mindset measure, this specific item exhibited the highest factor loadings on the common factor alongside the strongest multi-item scale correlation, demonstrated robust and theoretically consistent criterion validity, and provided the most conceptually straightforward indicator available for large-scale assessment (Rammstedt et al., 2022).

#### Resilience

Resilience was measured with three components: self-efficacy, perseverance, and sense of meaning in life.

##### Self-efficacy

Participants were asked to report the extent to which they agreed with five statements about their general sense of efficacy, particularly in the face of adversity (e.g., “My belief in myself gets me through hard times.”, “I feel that I can handle many things at a time.”). All items were measured on a 4-point Likert scale ranging from 1 (*strongly disagree*) to 4 (*strongly agree*), with higher scores indicating stronger beliefs in one’s ability to overcome challenges and achieve goals. The Cronbach’s alpha in this sample was 0.78.

##### Perseverance

Perseverance was measured by three items (e.g., “I find satisfaction in working as hard as I can.”, “Once I start a task, I persist until it is finished.”). All the items were measured on a 4-point Likert scale ranging from 1 (*strongly disagree*) to 4 (*strongly agree*), with higher scores indicating a higher level of persistence and motivation to complete the tasks. The Cronbach’s alpha in this sample was 0.77.

##### Sense of meaning in life

Sense of meaning in life was measured by three items (e.g., “My life has clear meaning or purpose.”). Each item was rated from *strongly disagree* (1) to *strongly agree* (4). Higher scores suggested a higher level of perceived meaning and purpose in life. The Cronbach’s alpha in this sample was 0.89.

### Student covariates

Gender, race, language spoken at home, and socioeconomic status were included as control variables. Specifically, gender was dummy coded as 0 = women and 1 = men. Race was dummy coded as four racial/ethnic groups with three dummy variables (i.e., White, Black/African American, Hispanic/Latino, and others). The language spoken at home was dummy coded as 0 = English and 1 = language other than English. Socioeconomic status was operationalized using the index of economic, social, and cultural status (ESCS) in the PISA dataset, which was derived from students’ reports on their parents’ education, their parents’ occupation, and their household items and other possessions at home (OECD, 2019).

### Data analyses

Data analysis was conducted using SPSS Version 29.0 and *M*plus 8.11 (Muthén and Muthén, 1998–2017). Initial analyses examined descriptive statistics, including means, standard deviations, and bivariate correlations. The skewness and kurtosis values were examined to evaluate the normality of the data distribution. According to George and Mallery (2019), values within the range of −2 to 2 confirmed a normal distribution. To examine differences among participants on these three covariates, independent samples *t*-tests were conducted for gender and language spoken at home, and a one-way ANOVA was conducted for race. The results revealed significant differences in the outcome variables across gender, language spoken at home, and race. Thus, all were included as control variables in subsequent analyses. As the data has a nested structure, the assumption of independent observations is violated. To account for the nested structure of the PISA data (e.g., students clustered within schools) and reduce the risk of statistical deflation of standard errors, structural equation modeling was conducted using the design-based complex survey approach (TYPE = COMPLEX) in *M*plus. The school identification variable was specified as the cluster identifier. This approach utilizes a cluster-robust Huber-White sandwich estimator to adjust standard errors and chi-square test statistics for non-independence within clusters. Therefore, standard errors, and corresponding statistical significance levels reported in this study are adjusted for nested data structure.

Structural equation modeling was conducted to examine the relations among variables of interest. To further examine the significance of the indirect effects, bootstrapping with a total of 5000 bootstrap samples was conducted. Missing data in the current dataset were 4.15%, which was less than 5% and thus inconsequential (Schafer, 1999). Full information maximum likelihood (FIML) estimation was adopted to handle the missing data. Survey weights were used to ensure the calculation of appropriate estimates of population parameters and their sampling error. Using weights also allows for valid estimates and inferences of the population based on the individual students selected for the study (OECD, 2019). The following indices were evaluated: Chi-square, comparative fit index (CFI), Tucker-Lewis index (TLI), root mean square error of approximation (RMSEA), and standardized root mean square residuals (SRMR). The CFI values > 0.95, TLI values > 0.95 RMSEA values < 0.06, and SRMR < 0.08 indicate a good model fit (Hu and Bentler, 1999). In addition, to evaluate the construct validity, composite reliability (CR) and average variance extracted (AVE) were calculated. Construct reliability was considered robust if CR values exceeded 0.70, and convergent validity was evaluated against an AVE benchmark of 0.50 (Fornell and Larcker, 1981).

## Results

### Descriptive analyses

The mean, standard deviation, bivariate correlations, as well as the skewness and kurtosis values of the variables were presented in Table 1. Nearly all variables exhibited significant correlations with each other. All the skewness and kurtosis values fell within the range of −2 to 2, indicating that the data follow a normal distribution.

**TABLE 1:** Correlations, means, standard deviations, skewness, and kurtosis.

	TAS	TES	COOP	COMP	SE	PER	MEANING	MINDSET
TAS	–							
TES	0.34***	–						
COOP	0.23***	0.19***	–					
COMP	0.01	0.03*	0.13***	–				
SE	0.13**	0.17***	0.29***	0.18***	–			
PER	0.14**	0.19***	0.23***	0.15***	0.42***	–		
MEANING	0.14***	0.17***	0.26***	0.05**	0.46***	0.30***	–	
MINDSET	0.08***	0.05***	0.010	0.05**	0.05***	0.05***	−0.02	–
M	3.14	2.86	2.55	2.81	3.08	3.11	2.85	2.88
SD	0.77	0.75	0.68	0.70	0.50	0.58	0.78	0.95
Skewness	−0.64	−0.68	0.06	−0.08	−0.24	−0.37	−0.68	−0.45
Kurtosis	−0.49	0.34	−0.09	−0.43	1.17	−0.25	1.12	−0.76

TAS, teachers’ academic support; TES, teachers’ emotional support; COOP, cooperation; COMP, competition; SE, self-efficacy; PER, perseverance; MEANING, sense of meaning in life; M, Mean; SD, Standard deviation. *p < 0.05; **p < 0.01; ***p < 0.001.

### CFA and SEM analyses

The CFA model showed a good model fit to the data: χ^2^ (278) = 1508.834, *p* < 0.001, CFI = 0.972, TLI = 0.967, RMSEA = 0.030 (90% CI [0.029, 0.032]), SRMR = 0.033. All factor loadings were larger than 0.50, with the majority higher than 0.70. All other parameter estimates as well as their standard errors were reasonable, indicating that the measurement model was robust to examine the structural relationships among the variables. The measurement model demonstrated strong construct validity (Supplementary Table 1). Latent construct reliability was supported, with composite reliability (CR) values ranging from 0.77 to 0.91. Convergent validity was firmly established for six of the seven multi-item constructs, values ranging from 0.53 to 0.74 (see Supplementary Table 2). Although the AVE for Self-Efficacy (SE) was 0.44, its CR was 0.79. According to Fornell and Larcker (1981), an AVE below 0.50 is acceptable if the composite reliability is well above 0.60.

The results of the SEM model indicated that the global model fit was good: χ^2^ (441) = 2345.942, *p* < 0.001, CFI = 0. 959, TLI = 0.953, RMSEA = 0.030 (90% CI [0.029, 0.031]), SRMR = 0.036. All parameter estimates and their associated standard errors were within reasonable ranges. The variances were positive and the standardized solutions did not exceed 1. No extremely large coefficients or standard errors were observed. The *R*^2^ of self-efficacy, perseverance, and sense of meaning of life were 0.18, 0.14, and 0.11, respectively. In terms of the three covariates, the results showed that gender differences only occurred for perseverance. Specifically, female students had higher perseverance than male students (β = −0.08, *p* < 0.001). In terms of race/ethnicity, Black students reported significantly higher self-efficacy (β = 0.08, *p* < 0.001) and meaning in life (β = 0.08, *p* < 0.001) compared to White students. In addition, other minority groups showed lower meaning in life (β = −0.05, *p* = 0.003) compared to White students. Regarding language spoken at home, there were no significant differences across the three dependent variables. For ESCS, it positively predicted self-efficacy (β = 0.08, *p* = 0.001), perseverance (β = 0.09, *p* < 0.001), and meaning in life (β = 0.04, *p* = 0.027).

**FIGURE 1:**
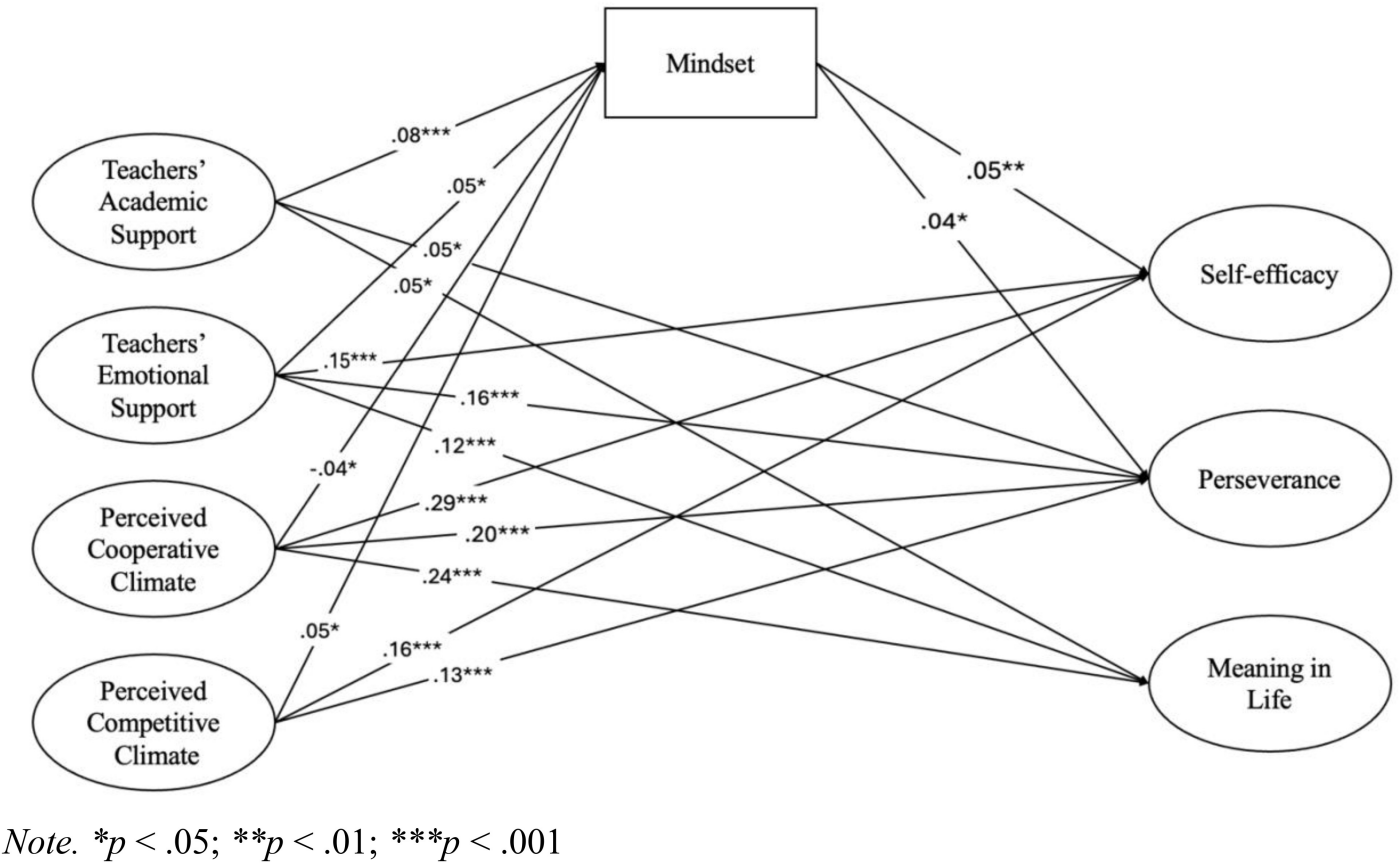
Structural equation model with standardized coefficients. *p < 0.05; **p < 0.01; ***p < 0.001.

After controlling for the covariates, the results of SEM (see Figure 1) indicated that teachers’ academic support was positively related to mindset (β = 0.08, *p* < 0.001) and two components of resilience: perseverance (β = 0.05, *p* = 0.014) and sense of meaning in life (β = 0.05, *p* = 0.027), but not self-efficacy (β = 0.01, *p* = 0.67). Teachers’ emotional support was positively related to mindset (β = 0.05, *p* = 0.017) and all three components of resilience (self-efficacy, β = 0.15, *p* < 0.001; perseverance, β = 0.16, *p* < 0.001; and sense of meaning in life, β = 0.12, *p* < 0.001). Perceived cooperative climate was negatively related to mindset (β = −0.04, *p* = 0.034), but positively predicted all three components of resilience: self-efficacy (β = 0.29, *p* < 0.001), perseverance (β = 0.20, *p* < 0.001), and sense of meaning in life (β = 0.24, *p* < 0.001). Perceived competitive climate was positively associated with mindset (β = 0.05, *p* = 0.015) and two components of resilience: self-efficacy (β = 0.16, *p* < 0.001), perseverance (β = 0.13, *p* < 0.001), but not sense of meaning in life (β = 0.03, *p* = 0.161). Mindset positively predicted self-efficacy (β = 0.05, *p* = 0.004) and perseverance (β = 0.04, *p* = 0.038), but not sense of meaning in life (β = −0.03, *p* = 0.082). The results of bootstrapping revealed that mindset only mediated the relation between teachers’ academic support and self-efficacy (β = 0.004, (95% CI [0.001, 0.008])).

A latent variable sensitivity analysis was conducted, which indicated that the structural paths and indirect effects remained robust to potential measurement error. Specifically, we specified a latent growth mindset factor (GM_L) and constrained its error variance using the item’s observed variance (0.894) across two reliability specifications: moderate (ρ = 0.60; error variance = 0.358; Hair et al., 2019) and conservative (ρ = 0.50; error variance = 0.447; Wanous et al., 1997). As shown in Supplementary Table 1, partitioning out measurement error deattenuated and strengthened the path coefficients while retaining statistical significance. These results demonstrate that single-indicator attenuation does not compromise the study’s conclusions; rather, controlling for measurement error reinforces the hypothesized structural and mediation mechanisms.

## Discussion

The present study examined the interactive effects of teachers’ support, perceived cooperative and competitive climates, and mindset on adolescents’ resilience. Overall, the findings of this study were consistent with previous studies (Baldrighi et al., 2011; Guo et al., 2020; Janssen and van Atteveldt, 2023), indicating that adolescents’ resilience was predicted by teachers’ support, their perceptions of peer cooperation, and beliefs about their own intellectual capacities. Notably, several novel findings regarding the complex interactions among the variables were revealed.

Specifically, the results from the model revealed positive relations between mindset and two components of resilience (i.e., self-efficacy and perseverance), which was consistent with previous research (Iqbal et al., 2021; Janssen and van Atteveldt, 2023; Yeager and Dweck, 2012). However, the findings indicated that mindset was not related to sense of meaning in life. One possible explanation is that placing excessive emphasis on adopting a growth mindset might lead adolescents to focus on continuous improvement in ways that might lead them to concentrate on what they lack and overlook their achievements, which leaves little time to reflect on the meaning of past and current experiences in a manner that promotes sense of purpose. Another possible explanation is that mindset, as measured in the current study, focused specifically on intelligence and thus may be more important for overcoming difficulties and challenges in the academic area than for developing an individualized sense of meaning and purpose in life, a process which likely involves seeking out opportunities and challenges beyond school.

The findings also demonstrated that teachers’ academic support (e.g., providing individualized attention, offering extra support when needed, and continuing to teach until students fully understand the content) directly and positively predicted adolescents’ mindset, perseverance, and sense of meaning in life. These findings not only align with previous research (Kang et al., 2024) indicating that teachers’ sustained academic support can promote adolescents’ growth mindset and perseverance, but also demonstrate the novel impact of teachers’ academic support on students’ sense of meaning in life. One potential reason is that when teachers provide academic support, they reinforce students’ efforts and help students to set goals and navigate challenges. This guidance may enable students to explore their interests and passions within the school context and to understand how their efforts align with broader meaning and purpose.

Additionally, teachers’ emotional support demonstrated positive relations with mindset and all three resilience components. These findings provide empirical evidence demonstrating the positive role of teachers’ emotional support in facilitating students’ growth mindset. This positive connection may stem from the fact that emotional support from teachers helps create a safe and nurturing learning environment. In such environments, students feel psychologically and emotionally safe, which makes them more willing to embrace challenges and reframe mistakes and failures as opportunities for growth (Johnson et al., 2020). Additionally, the positive association between emotional support and resilience suggests that when teachers show their understanding and display genuine interest in adolescents’ thoughts and opinions, they can foster students’ capacity to overcome stress and adversity, consistent with prior findings (Ji et al., 2023). Notably, emotional support showed stronger effect sizes than academic support across all resilience components, which highlights the crucial role of teachers’ emotional support in facilitating adolescents’ resilience.

The results also highlighted the role of perceived cooperative and competitive climate in predicting resilience. Perceived cooperative climate showed the largest effect size among all predictors, demonstrating the importance of supportive peer relationships for adolescent resilience (Baldrighi et al., 2011). This finding is consistent with previous research suggesting that when students perceive their peers as mutually supportive, they tend to develop stronger social-emotional bonds and support networks that buffer against adversity (Huang and Lajoie, 2023). Additionally, cooperative school climates may foster prosocial behaviors such as helping others, which may enhance students’ sense of meaning in life by allowing students to feel they are making a positive impact on others’ lives (Xi et al., 2017).

Interestingly, when it came to the relations between perceived cooperative climate and mindset, we found that a perceived cooperative climate negatively predicted mindset. Because these findings were unexpected, and because relatively little empirical work has examined the relations between competitive versus cooperative classroom environments with adolescents’ mindset views, we wish to exercise caution in exploring possible explanations. One possible explanation is that, because individuals with stronger growth mindsets show greater preference for cooperative educational environments (Gocłowska et al., 2017), students in the current study with stronger growth mindsets may have perceived their environment as less cooperative, due to the environment not meeting their desired levels of collaboration. Because adolescents engage in social comparison more heavily than younger children (Wigfield and Eccles, 1994), a strongly cooperative climate may increase the visibility of relative ability differences among peers and reinforce fixed mindset beliefs rather than mitigate them. In addition, the norms of cooperative climates can in some cases undermine peer support for high performers (Hendricks et al., 2023), which might discourage the desire for personal growth reflected in growth mindset. Another possible factor is the difference in cultural orientations toward individualism and collectivism (Hofstede, 2001), which may influence how students interpret and experience cooperation. Specifically, in more individualistic contexts, cooperative climate can expose status differences and threaten students rather than scaffold performance (Webb, 1989). This finding can also be understood through attribution theory (Weiner, 1985), which suggests that in cooperative environments, students are more likely to attribute academic outcomes to collective group effort rather than individual ability development. Because growth mindset is primarily a belief about the malleability of one’s own intelligence through personal effort and strategy (Dweck and Yeager, 2019), attributing outcomes to group processes may reduce the perceived relevance of individual growth-oriented beliefs.

Regarding perceived competitive climate, this variable was positively associated with mindset, self-efficacy, and perseverance, but not sense of meaning in life. The positive associations with self-efficacy and perseverance are consistent with prior research demonstrating that competitive environments can motivate students to develop confidence in their abilities and sustain effort toward academic goals (Chen and Chiu, 2016; Chen et al., 2025; Kim et al., 2023). However, perceived competitive climate was not related to a sense of meaning in life. The absence of a significant association with sense of meaning in life might be interpreted through the lens of self-determination theory (Ryan and Deci, 2000). Specifically, while competitive environments may satisfy students’ need for competence, they are less likely to address the deeper psychological need for purpose and meaningful connection, suggesting that competitive climate alone is insufficient to foster this dimension of resilience. The findings also indicate that cooperative and competitive climates were not necessarily mutually exclusive. Students can perceive their schools as mixed competitive-cooperative environments, and the mixture of both climates may contribute positively to adolescents’ development (Lee et al., 2022).

The findings regarding indirect effects indicated that teachers’ academic support predicted adolescents’ self-efficacy through mindset. Consistent with Bandura’s (2001) SCT theoretical framework, environmental factors can influence resilience capabilities through individual-level factors. Specifically, while teachers’ academic support did not directly predict self-efficacy, it can foster a growth mindset, which in turn enhances self-efficacy. These findings suggest that teachers’ academic support plays a multifaceted role in promoting adolescents’ resilience through both direct and indirect pathways. By providing instrumental assistance and resources, teachers cultivate students’ beliefs in their ability and their intrinsic drive to develop competence, which ultimately strengthens their overall resilience.

### Implications

The findings yield several key implications. First, while both academic support and emotional support enhance adolescents’ resilience, emotional support emerges as a stronger predictor. Thus, teachers should prioritize creating supportive emotional connections through active listening, connecting with students’ interests and goals, and creating an inclusive environment to help students feel valued and understood. Additionally, teachers’ academic support could also boost students’ resilience through mindset. Thus, giving constructive feedback and offering extra tutoring if needed are necessary, especially when adolescents are struggling with academic work. Second, teachers should thoughtfully blend cooperative and competitive elements. Specifically, teachers could include structured group projects and peer tutoring to promote peer communication and mutual learning. Additionally, integrating healthy challenges, such as low-stakes quiz competitions, informal debates, and contests could also build adolescents’ resilience. Third, since growth mindset could promote adolescents’ beliefs in their abilities and their persistence to accomplish their goals, teachers can implement classroom practices to promote growth mindset by providing feedback that highlights students’ hard work and effective learning strategies, acknowledging students’ progress in scaffolded tasks, and encouraging students to share their stories of overcoming difficulties. This comprehensive approach could help adolescents to build self-confidence in their educational journeys, which would further build their long-lasting resilience.

### Limitations

The findings of the current study should be interpreted within the context of several limitations. First, while three primary components of resilience were examined, other subdimensions of resilience (e.g., emotional regulation, help-seeking behaviors; Cassidy, 2016) should be considered in future research to provide a more comprehensive understanding of resilience mechanisms. Second, the assessment of mindset depended on a single item, which might potentially explain the small effect size observed in the relations between mindset and other variables of interest. Because single item measures cannot adequately capture the multidimensional nature of growth mindset, future studies would benefit from including multiple items to assess mindset, which could provide a more nuanced examination of its connection to other variables. Third, despite the large sample size in the current sample, the cross-sectional nature of the data cannot support cause-and-effect relations among the variables of interest. Thus, experimental and longitudinal approaches are needed to further explore possible causal effects.

## Conclusion

The current study examined the interactive roles of teachers’ academic and emotional support, perceived cooperative and competitive climates, and mindset in predicting adolescents’ resilience. The findings revealed that all the predictors positively contributed to adolescents’ mindset and resilience, except for the negative association observed between perceived cooperative climate and mindset. Additionally, teachers’ academic support positively impacted resilience through mindset. Such interactive relations underscore the importance of adopting a holistic approach that integrates both academic and emotional support to cultivate a growth mindset in adolescents, which in turn enhances adolescents’ abilities to thrive despite difficulty. Furthermore, creating an environment that strikes a balance between collaboration and healthy competition can empower adolescents to navigate adversity and build capabilities to overcome the challenges of this developmental stage.

## Data Availability

Publicly available datasets were analyzed in this study. This data can be found here: The data that support the findings of this study are publicly available from the Organization for Economic Co-operation and Development (OECD) Programme for International Student Assessment (PISA) at https://www.oecd.org/pisa/data/.
