# Supplementary material for: Building adolescents’ resilience: teachers’ support, perceived cooperative and competitive climates, and mindset
Source: Front Psychol. 2026 Jul 29;17:1897482. doi: 10.3389/fpsyg.2026.1897482 (PMC13461312; doi:10.3389/fpsyg.2026.1897482)
Supplement: Supplementary file 1 [file Table_1.docx]

**Supplementary Table 1: Sensitivity Analysis Results**

| Structural Path / Indirect Effect | Manifest Model (Original) | Sensitivity Model A (*ρ* = .60) | Sensitivity Model B (*ρ* = .50) |
| --- | --- | --- | --- |
| Academic Teacher Support 🡪 Growth Mindset | $\beta$= .08*** | $\beta$ = .11*** | $\beta$ = .12*** |
| Emotional Support 🡪 Growth Mindset | $\beta$ = .05* | $\beta$ = .07* | $\beta$ = .07* |
| Perceived Cooperative Climate 🡪 Growth Mindset | $\beta$ = -.04* | $\beta$ = -.05* | $\beta$ = -.05* |
| Perceived Competitive Climate 🡪 Growth Mindset | $\beta$ = .05* | $\beta$ = .06* | $\beta$ = .07* |
| Growth Mindset 🡪Self-Efficacy | $\beta$ = .05** | $\beta$ = .07** | $\beta$ = .07** |
| Growth Mindset 🡪Perseverance | $\beta$ = .04* | $\beta$ = .05* | $\beta$ = .06* |
| TS 🡪 Growth Mindset 🡪Self-Efficacy | $\beta$ = .004* | $\beta$ = .007* | $\beta$ = .009* |
