# Supplementary material for: Building adolescents’ resilience: teachers’ support, perceived cooperative and competitive climates, and mindset
Source: Front Psychol. 2026 Jul 29;17:1897482. doi: 10.3389/fpsyg.2026.1897482 (PMC13461312; doi:10.3389/fpsyg.2026.1897482)
Supplement: Supplementary file 2 [file Table_2.docx]

**Supplementary Table 2: Psychometric Properties of Multi-Item Latent Variables**

| Latent Variable | Number of Items | Composite Reliability (CR) | Average Variance Extracted (AVE) |
| --- | --- | --- | --- |
| Teachers’ Academic Support (TS) | 4 | 0.90 | 0.70 |
| Teachers’ Emotional Support (ES) | 3 | 0.88 | 0.71 |
| Cooperation (COOP) | 4 | 0.91 | 0.72 |
| Competition (COMP) | 4 | 0.85 | 0.59 |
| Perseverance (PER) | 3 | 0.77 | 0.53 |
| Meaning in life (MEANING) | 3 | 0.90 | 0.74 |
| Self-Efficacy (SE) | 5 | 0.79 | 0.44 |
